# Hidden dangers-prevalence of blood borne pathogens, hepatitis B, C, HIV and syphilis, among blood donors in Sierra Leone in 2016: opportunities for improvement: a retrospective, cross-sectional study

**DOI:** 10.11604/pamj.2018.30.44.14663

**Published:** 2018-05-18

**Authors:** Emmanuel Edmond Yambasu, Anthony Reid, Philip Owiti, Marcel Manzi, Mariama Jeanne Sia Murray, Ama Kyerewaa Edwin

**Affiliations:** 1 Médicins Sans Frontières, Sierra Leone; 2 Operational Research Unit, MSF Brussels, Luxembourg; 3 Academic Model Providing Access to Healthcare (AMPATH), Eldoret, Kenya; 4 Medical Department, MSF Brussels, Luxembourg; 5 Child Health/EPI Programme-Ministry of Health and Sanitation, Sierra Leone; 6 Department of Psychological Medicine and Mental Health, School of Medicine, University of Health and Allied Sciences, PMB 31, Ho, Ghana

**Keywords:** HBV, HCV, HIV, syphilis, rural, urban, Sierra Leone, blood donors

## Abstract

**Introduction:**

Transmissible blood-borne infections are a serious threat to blood transfusion safety in West African countries; and yet blood remains a key therapeutic product in the clinical management of patients. Sierra Leone screens blood donors for blood-borne infections but has not implemented prevention of mother-to-child transmission for hepatitis B. This study aimed to describe the overall prevalence of hepatitis B and C, HIV and syphilis among blood donors in Sierra Leone in 2016 and to compare the differences between volunteer versus family replacement donors, as well as urban versus rural donors.

**Methods:**

Retrospective, cross-sectional study from January-December 2016 in five blood bank laboratories across the country. Routinely-collected programme data were analyzed; blood donors were tested with rapid diagnostic tests-HBsAg for HBV, anti-HCV antibody for HCV, antibodies HIV1&2 for HIV and TPHA for syphilis.

**Results:**

There were 16807 blood samples analysed, with 80% from males; 2285 (13.6%) tested positive for at least one of the four pathogens. Overall prevalence was: 9.7% hepatitis B; 1.0% hepatitis C; 2.8% HIV; 0.8% syphilis. Prevalence was higher among samples from rural blood banks, the difference most marked for hepatitis C. The proportion of voluntary donors was 12%. Family replacement donors had a higher prevalence of hepatitis B, C and HIV than volunteers.

**Conclusion:**

A high prevalence of blood-borne pathogens, particularly hepatitis B, was revealed in Sierra Leone blood donors. The study suggests the country should implement the prevention of mother-to-child transmission of hepatitis B and push to recruit more volunteer, non-remunerated blood donors.

## Introduction

Blood transfusions save lives and improve health but in low income countries, particularly in Africa, many patients requiring blood are faced with two crucial blood transfusion-related issues--blood shortages and unsafe blood [1]. In the World Health Organization (WHO) African blood safety and availability status survey reports, not much improvement was seen in many countries. A 2010 report showed the average annual blood donation rate as 4.3 units per 1000 population with a range from 0.2 per 1000 in Nigeria to 33.8 per 1000 in Mauritius. In 2013 the values were an average of 4.7 units per 1000 population with a range from 0.7 per 1000 in Nigeria to 39.9 per 1000 in Mauritius. In the same reports, Sierra Leone recorded 5.2 and 7.1 units per 1000 population for 2010 and 2013 respectively [2, 3]. This is very low compared to high-income countries whose mean donation rate is 36.4 donations per 1000 population with a range from 13.3-64.6 [4]. Many studies have shown a high prevalence of hepatitis B virus (HBV), hepatitis C virus (HCV), human immunodeficiency virus (HIV) and syphilis among blood donors in West African countries [5-10]. These infectious agents are serious threats to blood safety for the recipients and pose a public health problem. In 2001, the WHO African region adopted a strategy (document AFR/RC51/9) that aimed at improving blood transfusion safety and bridging the gap between blood needs and blood availability in health services. Its objectives were to recruit more low-risk donors to improve the safety of blood and blood products and to promote their appropriate use by clinicians. The strategy had defined targets: 100% of blood units transfused were to be screened for HIV and other blood-borne infections; and at least 80% of all donations were to be by volunteer, non-renumerated blood donors (VNRBD) [11]. In 2010, the WHO Africa Blood Safety Status Survey reported that out of 43 countries: 19 had attained 80-100% VNRBD status, seven had reached 50-79% and 17 were <50%. Similarly, in a 2013 report where 46 countries were surveyed, 22 had attained 80-100% VNRBD status, two reached 50-79%, and 22 were <50%. In the same reports, Sierra Leone attained 9.7% and 10% VNRBDs, 5.2 and 7.1 donations per 1000 population in 2010 and 2013 respectively [2,3].

However, Sierra Leone did screen donors for all the recommended pathogens, including HBV, HCV, HIV and syphilis. Blood remains a key therapeutic product in the clinical management of maternity and paediatric patients in Sierra Leone but often the demand is greater than the supply. The demand seems to be greater in the rural areas where malaria prevalence is higher and there are delays for referral of both maternal and paediatric cases from peripheral health units. There are three types of blood donors in Sierra Leone-VNRBD, family replacement donors (FRD) and paid donors. VNRBD provide their blood for free and usually on a regular basis. FRD are sought when a patient requires a transfusion yet there is inadequate or no blood in the bank. When these two sources fail, paid donors offer theirs for a negotiable fee. Studies of blood-borne pathogens in blood donors are limited in Sierra Leone. One was conducted in a small private mission hospital in northern Sierra Leone, which showed a high prevalence of HBV, HCV and HIV [12]. Similarly, studies have been conducted among patient sub-groups (pregnant women and general medical patients) that have shown high prevalence especially for HBV [13-15]. Despite the high prevalence of HBV reported in these studies, Sierra Leone has yet to implement the 24-48 hour post-delivery administration of hepatitis B vaccination plus hepatitis B immunoglobulin to babies born to HBsAg positive mothers, a strategy that has immensely reduced HBV infection elsewhere [16]. The country, however, does have hepatitis B as part of its vaccination schedule for under-five children starting from six weeks after birth. Reports show that VNRBD are the safest group of donors, as the prevalence of blood-borne infections is lowest among this group [9,17]. However, a study in Kiribati reported that both voluntary and family replacement donors had similar positive viral and/or bacterial serological test results [18]. It is unclear in Sierra Leone if differences exist in the prevalence of these infections between VNRBD and FRD, as well as urban and rural blood donors, aspects of blood donation safety that have not been explored. Thirty studies conducted in Ghana reported a higher prevalence of HBV in rural than urban settings [19]. This study thus aimed to describe the overall prevalence of the blood-borne infections (HBV, HCV, HIV and syphilis) among blood donors in selected regions in Sierra Leone in 2016 and to compare differences between VNRBD versus FRD and those in urban versus rural donors.

## Methods

**Design**: This was a retrospective, cross-sectional study conducted between June and August 2017.

**General setting** Sierra Leone is located in West Africa and has a population of 7.1 million, of whom 79% live in rural areas [20]. It borders on the Atlantic Ocean to the west, Guinea to the north and east, and Liberia to the south. The already weak and crippled health care system following the eleven year civil war (1991-2002) was hit by the world's worst Ebola epidemic from mid-2014 through early 2016, killing 3,956 people out of 14,124 reported cases as reported in WHO Ebola situation report 27^th^ March 2016 [21].

**Specific setting**: This study was carried out in two urban and three rural hospital blood bank laboratories, all of which are government-owned and operated. These five blood bank laboraties were selected because they were located in the five biggest hospitals covering the Eastern, Southern, Northern and Western regions of the country. “Urban” refers to laboratories in the capital city of Freetown while “rural” refers to those in the provinces of Bo, Kenema and Makeni. The two urban laboratories are the Connaught Blood Bank Lab and the Princess Christian Maternity Hospital (PCHM). The Connaught Hospital is the biggest in the country and is the central tertiary referral and teaching hospital. The PCMH Blood Bank Laboratory is located in the eastern part of Freetown and it serves both the tertiary referral maternity hospital (PCMH) and the Ola During Children's Hospital, (ODCH). The three rural blood banks (Makeni, Bo and Kenema) act as provincial laboratories serving the rest of the regions. The laboratory staff registers blood donors, screens them, takes blood, and records their information in paper registers designed by the national blood bank service. All blood banks obtain blood from three sets of donors: voluntary blood donors, family replacement blood donors; and commercial blood donors. Note that in Sierra Leone, FRD and paid donors are registered as FRD in the laboratory records.

**Screening for pathogens**: Screening of potential blood donors for blood-borne pathogens follows these steps: donations are not taken from those <15 years of age and >65 years, pregnant women, women on their menstrual period, or those who have received a recent blood transfusion (less than two weeks). Before blood is taken, donors are screened using rapid diagnostic test (RDT) strips from Fortress Diagnostic Limited, United Kingdom. The strips detect HBsAg for HBV, antibodies anti-HCV for HCV, HIV1&2 antibodies for HIV1&2, Treponema pallidum haemaglutination assay (TPHA) for Syphilis, Malaria and Haemoglobin. Approximately, three mililiters of blood is taken from the potential donor and then tested simultaneously for all pathogens. The test kits and methods of testing were similar in all blood banks.

**Interpretation of test results**: All test kits are RDT strips for HBV, HCV, HIV and syphilis. *Negative test*: only one pink coloured band appears on the control (C) region. *Positive test*: in addition to a pink coloured control (C) band, a distinct pink coloured band also appears in the test (T) region. *Invalid test*: a total absence of colour in both regions. Any positive or inconclusive results or a haemoglobin <10g/dl automatically lead to rejection of a donor. Malaria positive donors are allowed to donate, with the blood labeled “malaria positive” and used only in emergencies (acute blood shortage) and for adult patients only. Laboratory officers inform clinicians when dispensing this malaria-infected blood and the recipient will be given anti-malarial medication at the same time. HIV positive donors are linked to the HIV programme while no specific care is provided for those tested positive with hepatitis B, C and syphilis.

**Study population**: All blood donors registered and screened in the five blood bank laboratories between January 1 and December 31, 2016 were included in the study. As all the subjects in the study period were included in the study, no sample size was calculated.

**Data variables, collection and validation**: Data variables included name and location (urban or rural) of the laboratory, donor unique identifier, age and sex of donor, type of donor (volunteer or family replacement donor), type of pathogen tested and results. These variables were abstracted from the blood bank paper registers and double-entered and cross-checked in EpiData EntryClient version 4.0.1.44 (Epi Data Association, Odense, Denmark) by trained laboratory staff. The demographic information was recorded for each blood donor on arrival to the blood bank laboratory, after which the screening tests were performed. The laboratory supervisor collated and summarized both of these sources of data in the standard national blood bank monthly report and sent it to the central laboratory. The laboratories did not capture whether the donors were first-time or repeat donors.

**Analysis and statistics**: Prevalence was calculated based on the number of donors with positive results in a screening test divided by the number of donors tested. Data were analyzed and presented in terms of frequencies (and percentages) for categorical variables and the appropriate measure of central tendency (and dispersion) for continuous variables. Age was also re-grouped into 5-year age groups. We choose an age group division point from <28 and ≥28 years corresponding to population median point of 50% based on Sierra Leone's population and household census of 2015 [22]. Comparisons between different categorical groups used the Chi-square test and presented in odds ratios (95% confidence interval). Level of significance was set at <5%.

**Ethics approval**: The Sierra Leone Ethics and Scientific Review Committee granted approval to conduct the study. Similarly, the National Blood Bank Services and respective hospital in-charges granted their permission for the study. The study maintained confidentiality of data and subjects throughout. The study did not need to obtain informed consent from donors as the study was a document review, hence involved no direct engagement with donors. This research also fulfilled the exemption criteria set by the Médecins Sans Frontières Ethics Review Board for a posteriori analyses of routinely collected clinical data and thus did not require MSF ERB review. It was conducted with permission from the Medical Director, Operational Centre Brussels, Médecins Sans Frontières.

## Results

There were 16865 blood donors whose samples were tested at the five blood banks; 58 samples had missing results for all the four pathogens (HBV, HCV, HIV and syphilis) and therefore could not be included in the analysis; hence 16807 samples were analyzed. Table 1 shows their demographic characteristics with most being males (80%) and from the age range of 18-38 years. Of the donors, 2285 (13.6%) tested positive for at least one of the four pathogens. Overall, the prevalences of the blood-borne diseases were: 9.7% hepatitis B (1633/16803); 1.0% hepatitis C (159/16802); 2.8% HIV (473/16806); and 0.8% syphilis (133/16796). Table 2 shows the differences among the diseases by gender: there was a small but statistically greater prevalence of hepatitis B in males. Similarly, when comparing prevalence by age group (<28 vs ≥28 years), there was a small but statistically significant increase for syphilis in the older group, (0.6% vs 1%, p = 0.007). The prevalence of blood-borne diseases was higher among rural as compared to urban blood banks (Table 3) with the difference most marked for hepatitis C. Finally, Table 4 shows the differences in prevalence of the blood-borne diseases among family replacement and volunteer donors. FRD had higher prevalence of Hepatitis B, C and HIV compared to VNRBD.

**Table 1:** Demographic characteristics of blood donors in five blood bank laboratories in Sierra Leone in 2016

Characteristic		N	(%)
Sex	Male	13,426	(80)
	Female	3,362	(20)
Age (year)	Mean [standard deviation]	28.4	[7.3]
	Median [Interquartile Range]	27.0	[23-32]
	<18	18	(0.1)
	18 - <28	8,712	(52)
	28 - <38	5,796	(34)
	38 - <48	1,899	(11)
	48 – 55	237	(1.4)
	>55 (above donation age)	13	(0.1)
	*Missing age*	*132*	*(0.8)*

**Table 2:** Gender differences in prevalence of transmittable blood-borne diseases among blood donors in five blood bank laboratories in Sierra Leone in 2016

Disease	Total donors tested	Male		Female		Odds Ratio	(95% CI)	P-value
		N	(%)	N	(%)			
	16,788	13426	(80)	3362	(20)			
Any positive*	16,788	1,854	(14)	430	(13)	1.09	(0.98-1.22)	0.1
Hepatitis B	16,784	1355	(10)	277	(8)	1.25	(1.09-1.43)	0.001
Hepatitis C	16,783	124	(1)	35	(1)	0.89	(0.61-1.29)	0.5
HIV	16,787	368	(3)	105	(3)	0.87	(0.70-1.09)	0.2
Syphilis	16,777	106	(1)	27	(1)	0.98	(0.64-1.50)	0.9

* Positive for any of the four pathogens (hepatitis B, hepatitis C, HIV or syphilis)

HIV – human immunodeficiency virus

CI – confidence interval

**Table 3:** Differences between urban and rural prevalence of transmittable blood-borne diseases among blood donors in five blood bank laboratories in Sierra Leone in 2016

Disease	Total donors tested	Rural		Urban		Odds Ratio	(95% CI)	P-value
		N	(%)	N	(%)			
	16,807	10846	(65)	5961	(35)			
Any positive*	16,807	1,729	(16)	556	(9)	1.84	(1.67-2.04)	<0.0001
Hepatitis B	16,803	1,213	(11)	420	(7)	1.66	(1.48-1.86)	<0.0001
Hepatitis C	16,802	142	(1)	17	(0.3)	4.64	(2.80-7.67)	<0.0001
HIV	16,806	362	(3)	111	(2)	1.82	(1.47-2.26)	<0.0001
Syphilis	16,796	105	(1)	28	(0.5)	2.07	(1.36-3.14)	0.0005

* Positive for any of the four pathogens (hepatitis B, hepatitis C, HIV or syphilis)

HIV – human immunodeficiency virus

CI – confidence interval

**Table 4:** Differences in prevalence of transmittable blood-borne diseases between volunteer and family replacement blood donors in five blood bank laboratories in Sierra Leone in 2016

Disease	Total donors tested	Family replacement donor		Volunteer donor		Odds Ratio	(95% CI)	P-value
		**N**	**(%)**	**N**	**(%)**			
	16,746	14,760	(88)	1,986	(12)			
Any positive*	16,746	2,070	(14)	205	(10)	1.42	(1.22-1.65)	<0.0001
Hepatitis B	16,743	1,477	(10)	147	(7)	1.39	(1.17-1.66)	0.0002
Hepatitis C	16,742	148	(1)	11	(0.6)	1.82	(0.98-3.36)	0.05
HIV	16,745	431	(3)	40	(2)	1.45	(1.05-2.03)	0.02
Syphilis	16,736	119	(0.8)	13	(0.7)	1.23	(0.69-2.19)	0.5

* Positive for any of the four pathogens (hepatitis B, hepatitis C, HIV or syphilis)

HIV – human immunodeficiency virus

CI – confidence interval

## Discussion

This is the largest study on the prevalence of transmissible blood-borne pathogens among blood donors in Sierra Leone; and probably the first study that investigated the differences in prevalence among VNRBD and FRD as well as urban versus rural blood donors. This study found the following levels of prevalence: HBV, 9.7%, was higher compared to studies in Ivory Coast, Ghana and Cameroon (3.6-9.6%); but lower than those in Eastern Ethiopia, Nigeria, Equatorial Guinea and Sierra Leone (Masanga hospital) (10.0-15.0%). HCV, 0.9% was higher than in studies in Ivory Coast and Ethiopia East (0.4-0.7%); but lower than those in Equatorial Guinea, Ghana, Nigeria, Cameroon and Sierra Leone (1.3-8.0%). HIV, 2.8% was higher than the 2016 national figure of 1.2% in Sierra Leone, and was also higher than Cameroon, Ivory Coast, Ethiopia (0.1-1.8%); but lower than in Equatorial Guinea, Ghana, Nigeria and Sierra Leone (4.9-7.8%). Syphilis, 0.8% was higher than in studies in Ivory Coast (0.3%) and Eastern Ethiopia (0.1%); but lower than in Cameroon (8.1%) and Equatorial Guinea (21.5%) [5-10,12]. The high rate of HBV could be associated with the fact that there is no national programme to address the prevention and treatment of HBV particularly for adults. Unlike HIV and syphilis infections, the Sierra Leone national Reproductive, Maternal, Newborn, Child and Adolescent Health (RMNCAH) policy May 2017 did not make provision for mother-child prevention of Hepatitis B infection. Even though the routine vaccination programme for children under-five includes hepatitis B, children irrespective of their mothers' HBV status can only have access to this vaccine when they are six weeks old. This is probably because the mothers are not routinely screened for HBV infection during their antenatal clinic visits. The age group <28 years in Sierra Leone is characterized by active socio-economic activities including schooling, early marriage hence teenage pregnancy, blood donation, and commercial sex work. Studies in Douala, Cameroon, Eastern Ethiopia and Biako, Equatorial Guinea reported that blood donors were more frequent in the 18-27 year age group [8-10]; this is similar in this study in which 52% of the participants were below 28 years. VNRBD account for 12% of the study participants, which was very similar to the 2010 and 2013 WHO of African Blood Safety and Availability Status survey reports: 9.7% and 10.0% respectively [2,3]. When compared to other African countries, Sierra Leone (at 12%) ranked as 3^rd^ lowest in Africa and the least in West Africa in the 2013 report [3].

Most importantly, the figure further indicates that Sierra Leone is far below the target (at least 80%) set by WHO [11]. FRD recorded slightly higher prevalence in all pathogens except for syphilis; this is consistent with previous studies conducted in Duala, Cameroon 2014 and Egypt 2014 [9,17]. However, this is in contrast to the study in Kiribati which reported that there was no difference between the two types of donors with regards to blood-borne infections [18]. The higher prevalence among FRD in general could be associated with the fact that VNRBD are recruited, monitored and followed up by the Sierra Leone Red Cross, unlike FRD who are recruited on an ad hoc basis from family members or paid donors. Unfortunately, since paid donors were included among the group of FRD, we could not differentiate between these two groups. Analysis of purely paid donors may have shown an even higher prevalence. These figures are supported by a June 2016 WHO report that regular voluntary unpaid blood donors are the foundation of a safe blood supply because they are associated with lower levels of infection that can be transmitted by transfusions, including HIV and hepatitis viruses [23]. The prevalence of all pathogens was slightly higher among rural blood donors compared to urban ones, which is in agreement with the studies in Ghana [19]. The reasons for this could be associated with two key factors-access to medical care, including diagnostics, is more limited in rural communities compared to urban ones in Sierra Leone. Secondly, the literacy level in rural areas, described as the percentage of the population that has never attended school (32.7%) is almost three times higher than in the urban areas (11.5%) [22]; this could make it more difficult for rural communities to understand health messages regarding transmission routes of diseases including blood-borne infections. Further studies aimed at determining the epidemiology of blood-borne infections among the general population will be of value in determining the population prevalence. Some of the key strengths of this study are: there was a large sample size and it covered all five regions in the country. Test kits, the method of testing samples, recording of information and data collection were standardized across all the laboratories. It also followed the STrengthening the Reporting of OBservational studies in Epidemiology (STROBE) guidelines as highlighted in the British Medical Journal (BMJ) 2007 [24]. However, the study had some limitations: it relied on routine programme data collected by the laboratory staff and therefore could not control for data quality at the data collection stage; commercial or paid donors could not be separated because they were not uniquely labelled; the registers did not specify whether the volunteer blood donors were first, second or subsequent blood donations; and the results from this study cannot necessarily be representative of the country as only five blood bank laboratories were studied.

## Conclusion

This study found a high prevalence of blood-borne pathogens among blood donors in five blood bank laboratories in urban and rural Sierra Leone; hepatitis B virus infection was most common. Family replacement donors (including paid donors) and rural donors had slightly higher prevalence levels than voluntary and urban donors. These findings have important implications for Sierra Leone and add to the literature on blood-borne pathogens in West Africa. There are also a number of operational implications from this study. First, with almost 14% of all donations having at least one pathogen, there was a significant percentage of blood being unsuitable for donation. This is a loss in a context of stretched blood resources. All efforts to reduce these diseases will benefit the blood supply. Second, although the prevalence of pathogens was higher among family replacement blood donors, the positivity rate of test results among volunteer donors, the group WHO has confirmed the safest blood donors, was concerning and therefore suggests that the recruitment criteria to be re-examined and where necessary modified to promote safer blood donations. Third, with HBV prevalence most common in Sierra Leone, it is paramount that the country implement the prevention of mother-child-transmission, a programme that has been so successful elsewhere [16]. Finally, there should be some way to link positive blood donors of HBV, HCV and syphilis to care services if at all possible.

### What is known about this topic

- One study in Sierra Leone using blood donors in a rural hospital which showed a high prevalence of blood-borne diseases;
- Two studies in patients seeking general healthcare services in hospital and showed a high prevalence of blood-diseases;
- Another study in pregnant women in an urban hospital found the prevalence of hepatitis B virus infection at 6.2%.

### What this study adds

- The prevalence of blood-borne infections could be higher in rural than urban settings;
- Although the prevalence of blood-borne infections is lower among volunteer/non-remunerated blood donors, family replacement donors (including paid donors) were used much more commonly;
- Given the high prevalence of hepatitis B, starting a neonatal immunization programme in Sierra Leone would be worthwhile.

## Competing interests

The author declare no competing interests.
